# Integrating microbial 16S rRNA sequencing and non-targeted metabolomics to reveal sexual dimorphism of the chicken cecal microbiome and serum metabolome

**DOI:** 10.3389/fmicb.2024.1403166

**Published:** 2024-07-19

**Authors:** Yongxian Yang, Fuping Zhang, Xuan Yu, Liqi Wang, Zhong Wang

**Affiliations:** Key Laboratory of Animal Genetics, Breeding and Reproduction in the Plateau Mountainous Region, Ministry of Education, Guizhou University, Guiyang, China

**Keywords:** chicken, sexual dimorphism, cecal microbiota, serum metabolomics, integrated omics

## Abstract

**Background:**

The gut microbiome plays a key role in the formation of livestock and poultry traits via serum metabolites, and empirical evidence has indicated these traits are sex-linked.

**Methods:**

We examined 106 chickens (54 male chickens and 52 female chickens) and analyzed cecal content samples and serum samples by 16S rRNA gene sequencing and non-targeted metabolomics, respectively.

**Results:**

The cecal microbiome of female chickens was more stable and more complex than that of the male chickens. *Lactobacillus* and *Family XIII UCG-001* were enriched in male chickens, while *Eubacterium_nodatum_group*, *Blautia*, unclassified_Anaerovoraceae, *Romboutsia*, *Lachnoclostridium,* and norank_Muribaculaceae were enriched in female chickens. Thirty-seven differential metabolites were identified in positive mode and 13 in negative mode, showing sex differences. Sphingomyelin metabolites possessed the strongest association with cecal microbes, while 11β-hydroxytestosterone showed a negative correlation with *Blautia*.

**Conclusion:**

These results support the role of sexual dimorphism of the cecal microbiome and metabolome and implicate specific gender factors associated with production performance in chickens.

## 1 Introduction

Production performance in poultry displays a sex bias for growth rate, slaughter performance (increased muscle and decreased abdominal fat), feed conversion efficiency, and mineral utilization ability, and this dimorphism is of great practical significance for farmers (Rose et al., 1996; Lumpkins et al., 2008; Cui et al., 2021; Tumova et al., 2021). The chicken gut microbiome plays a significant role in nutrient processing including dietary fiber conversions to increase nutrient availability and thus contributes to host homeostasis (Wen et al., 2021). There is also clear evidence that the gut microbiota contributes to these sex-linked differences in production performance (Lee et al., 2017; Cui et al., 2021). Thus, gut microbiome profiles may provide clues to the regulation of the biological processes within the gut that discriminate between male and female chickens, and this information could be useful in designing more efficient rearing strategies to maximize production performance (Lee et al., 2017).

Previous studies have demonstrated that gender is an important factor that affects the composition and structure of gut microbiomes for humans and animals. In addition, different bacterial ecosystems have been identified in male and female chickens (Cui et al., 2021). For instance, Cobb 500 broiler chicks displayed <30% gender similarities in gut microbiota composition as early as 3 days post-hatch (Lumpkins et al., 2008), and gender-specific taxons included *Bacteroides*, *Megamonas*, *Megasphaera,* and *Phascolarctobacterium* for male chickens and *Akkermansia* for female chickens (Cui et al., 2021). Another study indicated that *Clostridium* and *Shigella* were more abundant in female chickens, while the indigestible fiber-degrading genus *Bacteroidetes* was more abundant in male chickens (Lee et al., 2017). However, although numerous studies have identified sex-linked differences in chicken gut microbiomes, the underlying mechanisms remain poorly understood.

Human and animal studies have indicated that gut microbiome sexual dimorphism is primarily the result of host metabolites and primarily steroid hormones and bile acids (Ervin et al., 2019; Sui et al., 2021). Sex hormones affect gastrointestinal motility, which in turn would affect the transit time through the gut and account for these differences (Cross et al., 2018). For example, the gut microbiomes of boars and sows raised together are significantly different, and castration (and thus androgen levels) resulted in the absence of sex-linked differences (He et al., 2019). The bile acid pool and the rate of bile acid synthesis also are higher in female chickens (Bennion et al., 1978) and bile acids that enter the hindgut can affect the composition of the gut microbiome and even inhibit the growth of specific microbes and significantly alter microbiome compositions between genders (Floch et al., 1972). Nevertheless, sex hormones and bile acids account for only a small proportion of metabolites in the serum metabolome and complicate screening that can then be correlated with gut microbiome populations (Tian et al., 2023). However, metabolomics technology now allows high-throughput analysis of a large number of metabolites with high sensitivity but has not been previously utilized to make these types of correlations (Liu et al., 2022).

If sex-dependent microbiota differences underlie the differences in sex-specific production performance, it might open new venues for designing effective strategies to improve chicken performance by manipulating microbiota and associated serum metabolome in a sex-specific way (Elderman et al., 2018). We hypothesized that differences in cecal microbiota between different gender chickens were related to different biological processes such as metabolite secretion. Therefore, in this study, we investigated the relationship between sex differences in serum metabolomes and microbiomes in healthy chickens. A total of 106 chickens of different genders were selected as research materials, and cecal content and serum samples were collected. We integrated microbial 16S rRNA gene sequencing and non-targeted metabolome detection technology to characterize and explore their interrelationships based on sex. The results can provide new insight for explaining differential production performance by gender in chickens and contribute to the development of sex-specific feeds to maximize production.

## 2 Materials and methods

### 2.1 Ethics statement

All animal studies were conducted according to the guidelines for the care and use of experimental animals established by the Ministry and Rural Affairs of the People’s Republic of China. The project used for this animal experiment was approved by the Animal Care and Use Committee at Guizhou University, China (approval number: EAE-GZU-2022-T050).

### 2.2 Animals and sample collection

The chickens used in these experiments were raised at the Scientific Research Chicken Farm of Guizhou University from June 2022 to October 2022. A total of 106 chickens were collected including 51 Guizhou yellow chickens (25 male chickens and 26 female chickens) and 55 Wumeng Black-Bone chickens (29 male chickens and 26 female chickens). Guizhou Yellow broiler is a Chinese hybrid line (Weining ♀ × New Hampshire × Plymouth Rock ♂) that possesses high market weights and tender meat, while Wumeng Black-Bone chicken possesses black tissues and bones. They are both meat-type chicken and are popular with consumers. The hatching batches, feeds, feeding methods, and management conditions were consistent. Specifically, chickens were fed in the same house, and cages were constructed of three-layer iron. The stocking density was 16 chickens from 0 to 4 weeks (male and female chickens mixed, not gendered); 8 chickens from 4 to 10 weeks (4 male and 4 female chickens mixed); and one chicken from 10 to 18 weeks. The chicken house temperature control was regulated using roller shades. The daily lighting time was 16 h, and the temperature ranged from 15 to 35°C. The chickens were fed with commercial feed twice a day at 9 AM and 5 PM, with access to food and watered *ad libitum*. Chickens were vaccinated according to routine immunization procedures for Marek’s and Newcastle disease, infectious bronchitis, bursal virus, and avian influenza. The chickens were weighed every 2 weeks with an electronic scale. No antibiotics were added within 1 month prior to cecal content sample collection.

**Figure 1:**
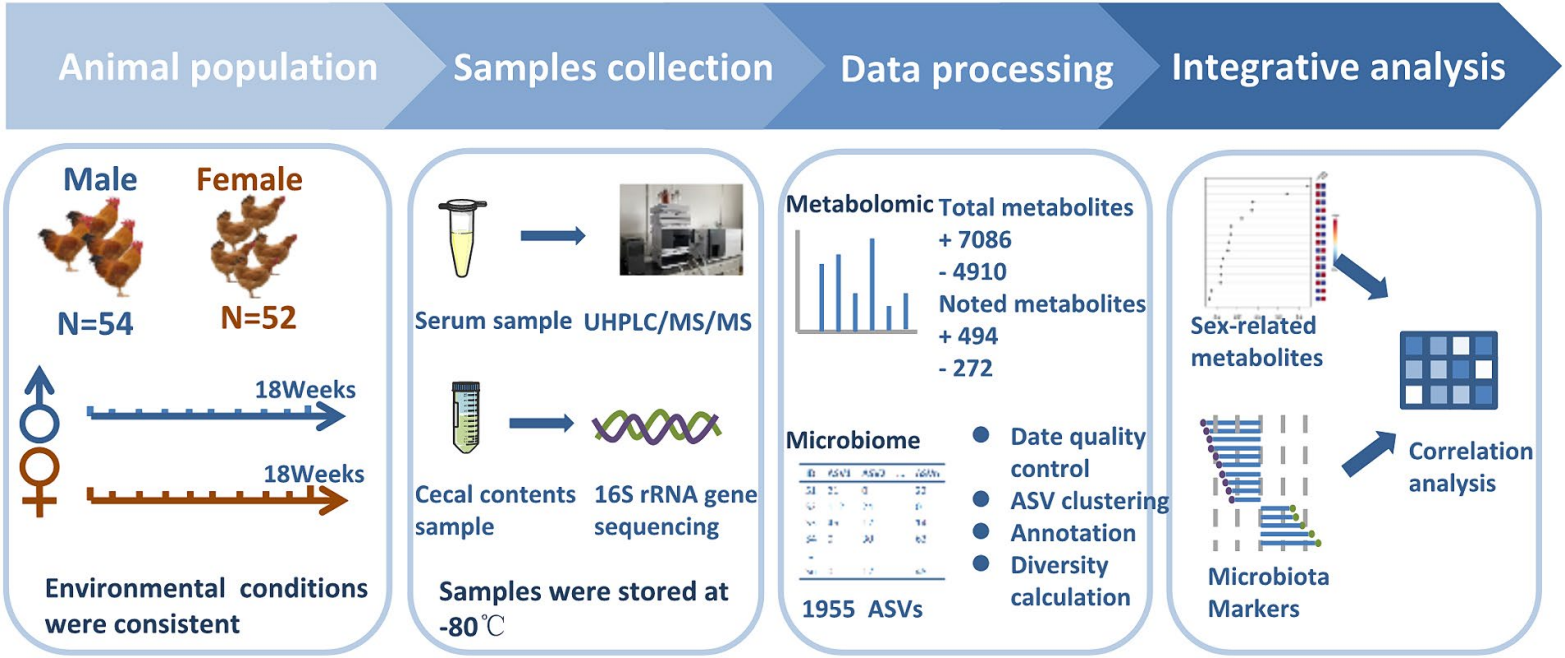
Experimental design. The experimental cohort comprised of 106 healthy chickens (male chickens *n =* 54, female chickens *n* = 52). Cecal content samples were collected at the age of 18 weeks and subjected to 16S rRNA gene sequencing to infer microbial profiles. Concurrent blood samples were collected to measure the non-targeted metabolome. Sexual dimorphism in chickens was explored after data pre-treatment of cecal microbiota and serum metabolome.

Since local chickens in these areas are generally 18 weeks old, these chickens were slaughtered at 18 weeks of age in this study. Chickens were euthanized by CO_2_ asphyxiation referred to previously reported methods (Haetinger et al., 2021). Whole blood was collected from wing veins and then was left undisturbed at room temperature to clot. After that, the clot was removed by centrifuging at 1000 × g for 5 min in a refrigerated centrifuge to obtain serum. Approximately 2 g cecal contents were collected into a clean sterile plastic 2 mL Eppendorf tube. Samples were immersed in liquid nitrogen immediately and were stored at −80°C. Due to five samples that showed hemolysis, only 101 serum samples were obtained for metabolome analysis including serum samples from 50 Guizhou yellow (25 male chickens and 25 female chickens) and 51 Wumeng black-bone (26 male chickens and 25 female chickens) chickens. All 106 cecal content samples for 16S rDNA gene sequencing and 101 serum samples for non-targeted metabolomics were processed at Shanghai Majorbio Bio-pharm Technology (Majorbio, Shanghai, China) and Shanghai Applied Protein Technology (Aptbio, Shanghai, China), respectively. The experimental flow chart is shown in Figure 1.

### 2.3 Cecal microbiota DNA extraction, v3–v4 region sequencing of 16S rRNA gene, and data processing

DNA was extracted from cecal content samples using a Magnetic Soil and Stool DNA Kit (Tiangen, Beijing, China) following kit instructions. The concentration and purity of DNA were determined using UV spectroscopy with a NanoDrop 1,000 instrument (Thermo Fisher, Pittsburg, United States) and 0.8% agarose gel electrophoresis. Primers 338F (5’-ACTCCTACGGGAGGCAGCAG-3′) and 806R (5’-GGACTACHVGGGTWTCTAAT-3′) were used to amplify the V3–V4 region of the 16S rRNA gene as previously described (Huse et al., 2008; Caporaso et al., 2011). The annealing temperature was set at 55°C for 27 cycles. The amplicons were purified from agarose gels using an AxyPrep DNA gel extraction kit (Corning, Glendale, United States). Purified amplicons were pooled in equimolar amounts, and paired-end sequencing was performed on an Illumina MiSeq platform (Illumina, San Diego, USA) according to standard protocols. A two-step tailed PCR approach was used to construct the paired-end libraries (Miya et al., 2015). The first-round PCR amplified the target region using a region of interest-specific primer; the overhang adapter sequence was used in the second-round tailed PCR to add indices and adapter sequences. The constructed libraries were sequenced with NovaSeq 6,000 SP 500 Cycle Reagent Kit (Illumina, San Diego, USA) at Majorbio Bio-pharm Technology (Shanghai) Co., Ltd. Barcodes, primers, and low-quality and ambiguous sequences were filtered out with Trimmomatic software (version 0.33) (Bolger et al., 2014). Cut adapt (version 1.9.1) was applied to identify and remove primer sequences (Martin, 2011). Paired-end reads from the clean data sets were clustered into tags by FLASH (version 1.2.11) (Magoc and Salzberg, 2011). Tags were then clustered into amplicon sequence variants (ASVs) using DADA2 (Callahan et al., 2016). ASV taxonomic assignments were conducted by the RDP classifier (version 2.2) (Wang et al., 2007). ASVs were annotated in the database Silva^1^ (Quast et al., 2012).

### 2.4 Construction of microbial co-occurrence network

Relative abundance of ASVs >0.05% was selected to construct clustering co-occurrence networks. The SparCC algorithm (Friedman and Alm, 2012) was used to construct a bacterial co-occurrence network. Correlations between ASVs (nodes) were calculated based on relative abundance using the PCIT algorithm (Reverter and Chan, 2008), and the paired taxa with absolute sparse correlation coefficient > 0.35 were selected for the next network construction. Cytoscape (version 3.7.1) (Lopes et al., 2010) was employed to evaluate the topological characteristics of co-occurrence networks and visualize the network. The stability of the network was represented by the percentage of negative interactions (competition) (Coyte et al., 2015; Hernandez et al., 2021). The complexity was calculated using the average number of lines connecting each node (Bader and Hogue, 2003).

### 2.5 Chromatography–mass spectrometry analysis of serum sample and metabolome data preprocessing

#### 2.5.1 Serum extraction

Serum samples from chickens were thawed at 4°C, and 100 μL aliquots were added to 400 μL of methanol/acetonitrile (1:1, v/v) to remove proteins, then vortex-mixed and cryogenically sonicated for 30 min, and then centrifuged for 20 min at 14000 × g at 4°C. The supernatant was dried in a vacuum centrifuge and dissolved in 100 μL acetonitrile/water (1:1, v/v) and centrifuged, and the supernatant was transferred to a sample vial for liquid chromatography-tandem mass spectrometry (LC-MS/MS) analysis.

#### 2.5.2 Analysis conditions of chromatography–mass spectrometry

Non-targeted metabolomic analysis of serum samples from chickens was performed at an ultra-high performance liquid chromatography-quadrupole /orbitrap high resolution mass spectrometry (UHPLC-Q-Exactive Orbitrap MS) with the methods as previously described (Dunn et al., 2011; Cai et al., 2015; Wang et al., 2016). A Vanquish HPLC system (Thermo Fisher, Pittsburg, United States) with an LC BEH Amide column (2.1 mm × 100 mm, 1.7 μm) coupled to an Orbitrap MS Q Exactive HFX mass spectrometer (Thermo Fisher, Pittsburg, United States) was used for separations.

##### 2.5.2.1 UHPLC chromatographic conditions

The column temperature was set at 25°C, the flow rate was at 0.5 mL/min, and the injection volume was 2 μL. The mobile phase compositions were as follows: composition A: water (containing 25 mM each ammonium acetate and ammonia) and composition B: acetonitrile. A gradient elution program was used as follows for analyte separation: 0–0.5 min, 95% B; 0.5–7.0 min, B changes linearly from 95 to 65%; 7.0–8.0 min, B changes linearly from 65 to 40%; 8.0–9.0 min, 40% B. The samples were placed in the auto-sampler at 4°C during the entire analysis process. To avoid the influence caused by the fluctuation of the instrument detection signal, samples were continuously analyzed in random order. QC samples were inserted into the sample queue to monitor and evaluate the stability of the system and the reliability of experimental data.

##### 2.5.2.2 Mass spectrometry conditions

After samples were separated using UHPLC, mass spectrometry was performed using a Triple TOF 6600 mass spectrometer (SCIEX, Framingham, USA), and electrospray ionization (ESI) positive and negative ion modes were used for detection. The ESI source conditions were set as follows: ion source gas 1 (Gas1) as 60, ion source gas 2 (Gas2) as 60, curtain gas as 30, source temperature: 600°C, ion spray voltage floating ±5,500 V (positive and negative modes). In MS-only acquisition, the instrument was set to acquire over the m/z range 60–1,000 Da, and the accumulation time for TOF MS scan was set at 0.20 s/spectra. In auto MS/MS acquisition, the instrument was set to acquire over the m/z range 25–1,000 Da, and the accumulation time for product ion scan was set at 0.05 s/spectra. The product ion scan was acquired using information-dependent acquisition, with high-sensitivity mode using collision energy fixed at 35 V ± 15 eV, declustering potential at 60 V (+) and − 60 V (−), and isotopes within 4 Da, with 10 candidate ions to monitor per cycle.

#### 2.5.3 Metabolome data preprocessing

ProteoWizard MSConvert was used to convert the raw MS data into MzXML files, and the data were imported into XCMS software. Parameters used to pick peaks were as follows: centWave m/z = 10 ppm, peak width = c (10, 60), prefilter = c (10, 100). Parameters used to group peaks were as follows: bw = 5, mzwid = 0.025, minfrac = 0.5. Collection of algorithms of metabolite profile annotation was used for annotation of isotopes and adducts. In the extracted ion features, only the variables having >50% of the non-zero measurement values in at least one group were selected. Compound identification of metabolites was performed by comparing accuracy m/z value (<10 ppm) and MS/MS spectra with an in-house database established with available authentic standards.

### 2.6 Statistical analysis

QIIME 2 platform (Bolyen et al., 2019)^2^ was used to calculate the alpha diversity of cecal microbiota with the Chao1, Shannon, and phylogenetic diversity (PD) indices (Shannon, 1948; Chao, 1984; Faith, 1992). Other statistical analyses and result visualization were performed on the R V4.4.0 environment (R Core Team, 2022). The performance data were analyzed using the two-way ANOVA on SPSS (version 26.0). Bray–Curtis distances were calculated to compare the beta diversity of the cecal microbial community between male and female chickens using principal coordinate analysis (PCoA). Alpha-diversity differences between the two groups were analyzed using the Wilcoxon rank-sum tests. Permutational multivariate analysis of variance (PERMANOVA) was used to explore the effects of genders on the composition of cecal microbiota (McArdle and Anderson, 2001). The effect size of each factor (R2) of PERMANOVA was used to determine the contribution and significance of genders on cecal microbial composition, and the *p*-values were calculated based on 9,999 permutations. Linear discriminate analysis (LDA) coupled with effect size measurements (LEfSe) was performed online platform^3^ (Segata et al., 2011) to identify bacterial taxa that differed significantly between male and female chickens, and the threshold was set to LDA > 2.0 and *p-*value <0.05. Spearman’s correlation analysis was conducted on R with ‘cor.test’ function to compute Pearson’s correlation coefficients between serum metabolites and cecal microbiota.

The metabolome data were processed on the MetaAnalyst 5.0 online platform (Pang et al., 2021).^4^ The dataset was normalized by log_10_ transformation of the m/z values. After sum normalization, the processed data of the two groups were compared using orthogonal partial least squares discriminant analysis (OPLS-DA). The robustness of the model was evaluated with 7-fold cross-validation and response permutation testing. The variable importance in the projection (VIP) value of each variable in the OPLS-DA model was calculated to assess its contribution to the classification. The importance threshold for the impact values for metabolic pathway topology analysis was set at 0.10 (Wang et al., 2012).

## 3 Results

### 3.1 Animals and growth performance

**Figure 2:**
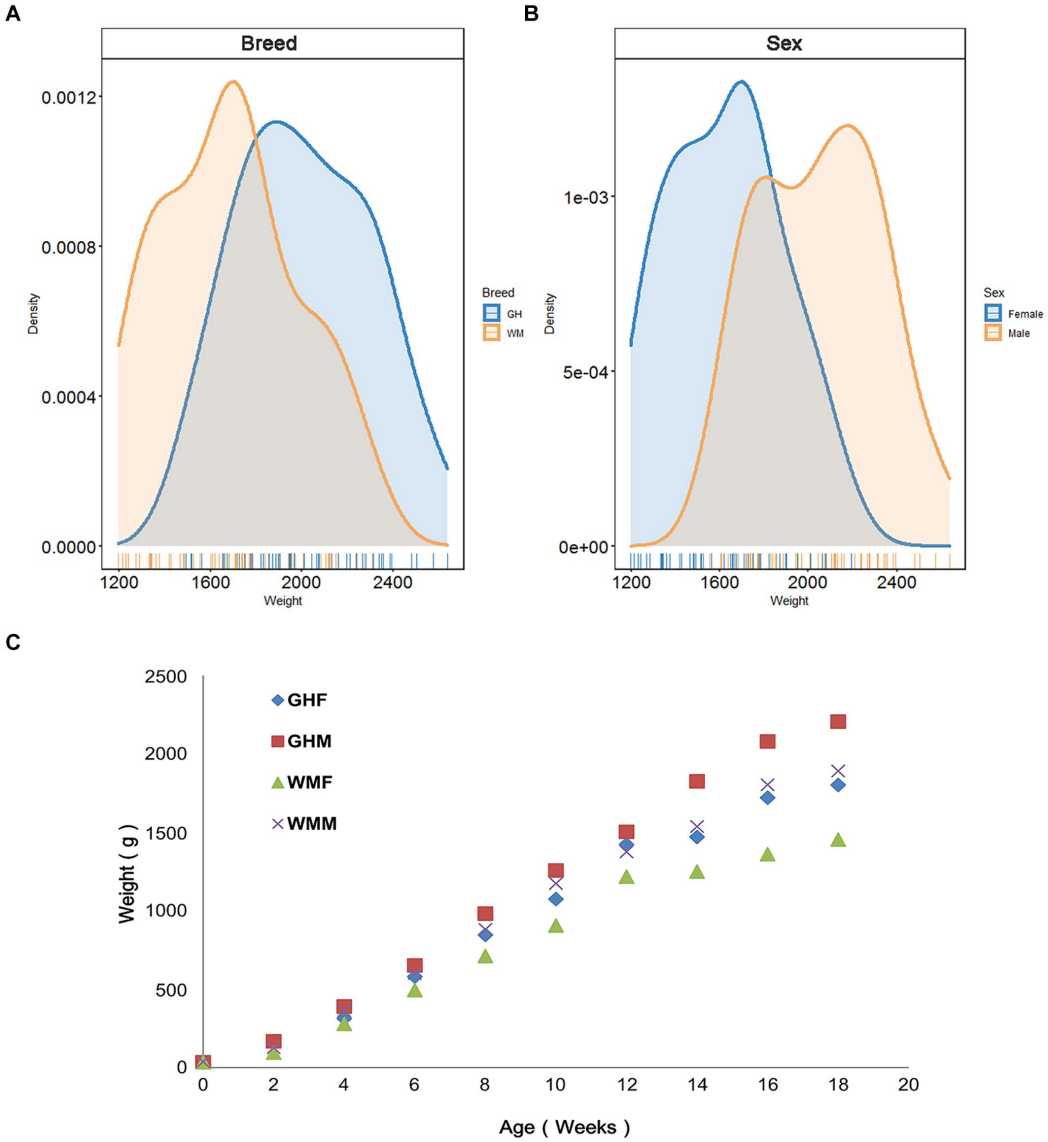
Body weights of experimental chickens. Comparison of 18-week-old body weight of chickens between different **(A)** breeds and **(B)** sexes. **(C)** Changes in body weight of experimental chickens from 0 to 18 weeks old. GH, Guizhou yellow chickens; WM, Wumeng Black-Bone chickens. The addition of F or M to breed designations indicates female and male chickens, respectively.

We utilized 106 chickens to examine whether breed factors significantly influenced production performance indicators and initially compared growth performance differences between Guizhou yellow chickens (GHC, *n* = 51) and Wumeng Black-Bone (WMC, *n* = 55) chicken breeds. We found that except for day 0 (*t*-test, *p* = 0.47) and week 4 (*p* = 0.077), GHC and WMC chickens significantly differed in their body weights (*p* < 0.05; Supplementary Table S1; Figures 2A C). The results of two-way ANOVA showed that there was no significant interaction between breed and sex for 18-week-age weights (*p* = 0.681; Supplementary Table S2). We then evaluated the role of gender on chicken growth performance and found that gender differences were significant for body weights (*p* < 0.01; Figure 2B). In particular, the body weights for the GHC male chickens (2205.92 ± 225.65 g, *n* = 25) were higher than body weights of GHC female chickens (1801.81 ± 191.62 g, *n* = 26) at the age of 18 weeks (Figure 2A), and the weights of WMC male chickens (1891.24 ± 220.36 g, *n* = 29) were also higher than WMC female chickens (1453.85 ± 189.00 g, *n* = 26).

### 3.2 Effect of gender on the composition of cecal microbiota in chickens

**Figure 3:**
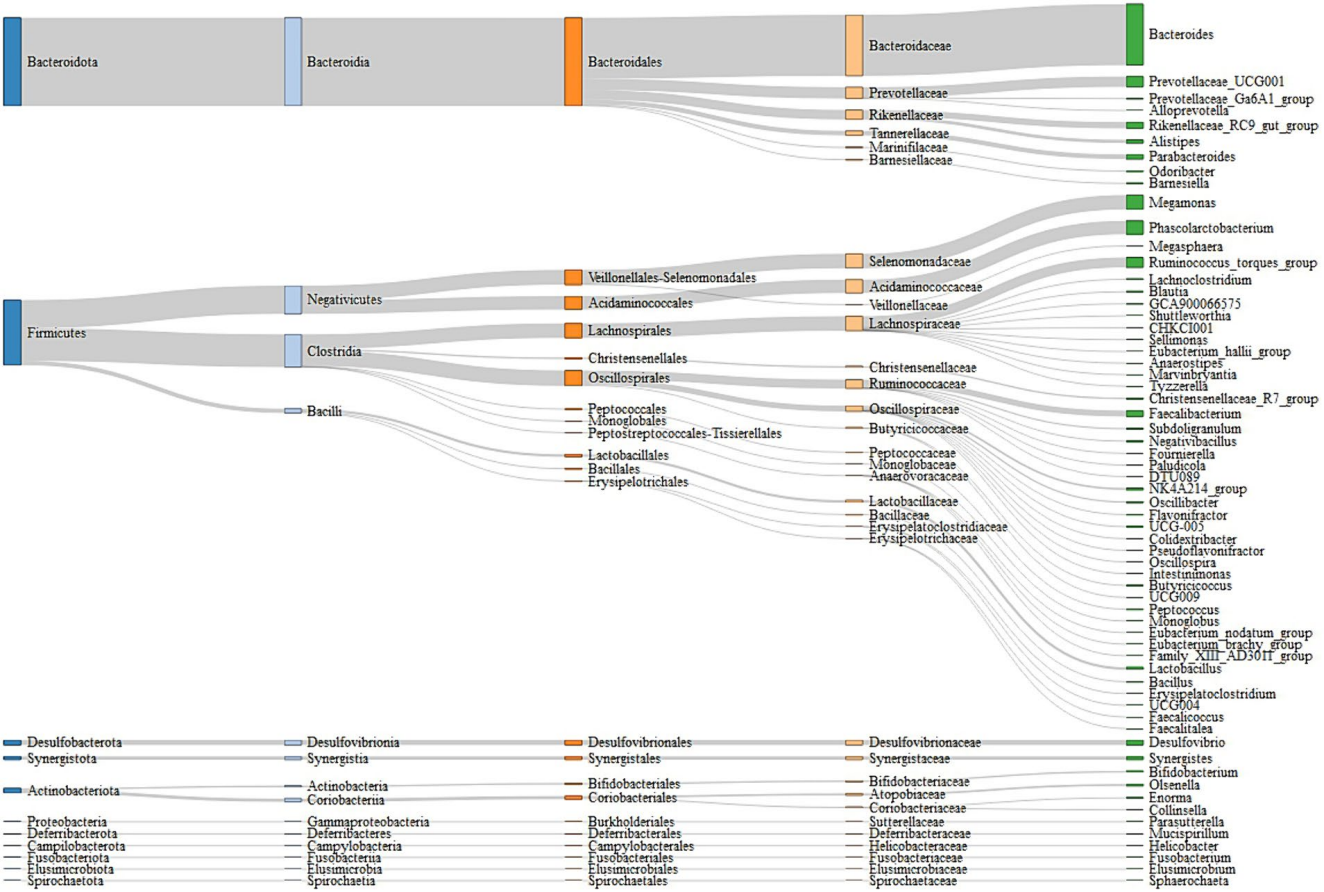
Cecal microbiota composition of experimental chickens depicted by a Sankey diagram.

A total of 5,087,788 high-quality reads were generated from 106 samples (47,998 reads per sample), and 1955 ASVs were clustered. Taxonomic assignments revealed that these ASVs could be classified into 16 bacterial and archaeal phyla, and 3 phyla were present in all samples. The mean relative abundance of 5 phyla was >1%, including Bacteroidetes (46.88%), Firmicutes (45.52%), Desulfobacterota (2.41%), Actinobacteriota (2.38%), and Synergistota (1.46%) (Supplementary Table S3). We identified 63 genera, and the predominant microbes in the ceca were *Bacteroides* (35.95%), *Megamonas* (8.48%), *Phascolarctobacterium* (7.87%), *Prevotellaceae_UCG-001* (6.09%), and *Ruminococcus_torques_group* (5.83%) (Figure 3; Supplementary Table S4). A permutation analysis for male chickens and female chickens showed that gender (*r* = 0.039, *p* = 0.002; Supplementary Figure S1) significantly affected the composition of chicken cecal microbiota. We further examined the structural characteristics of cecal microbiota interaction networks according to gender. A total of 252 and 273 ASVs were selected from cecal microbiota of the male and female groups, respectively, to construct microbial co-occurrence networks. The results showed that the network stability index for male and female chickens was 37.50 and 46.26%, respectively, and the complexity for female chickens was 7.01 and was 3.35-fold larger than that of the male microbial network (2.09) (Table 1).

**Table 1:** Indices of co-occurrence networks of cecal microbiota between male and female chickens.

Sex	Male chickens	Female chickens
Nodes	252	273
Edges	528	1913
Negative edges	198	885
Complexity	2.09	7.01
Stability (%)	37.50	46.26

Complexity, the average number of edges connected to each node; stability, the proportion of negative correlations to the total number of correlations.

### 3.3 Identification of gender-related cecal microbes

**Figure 4:**
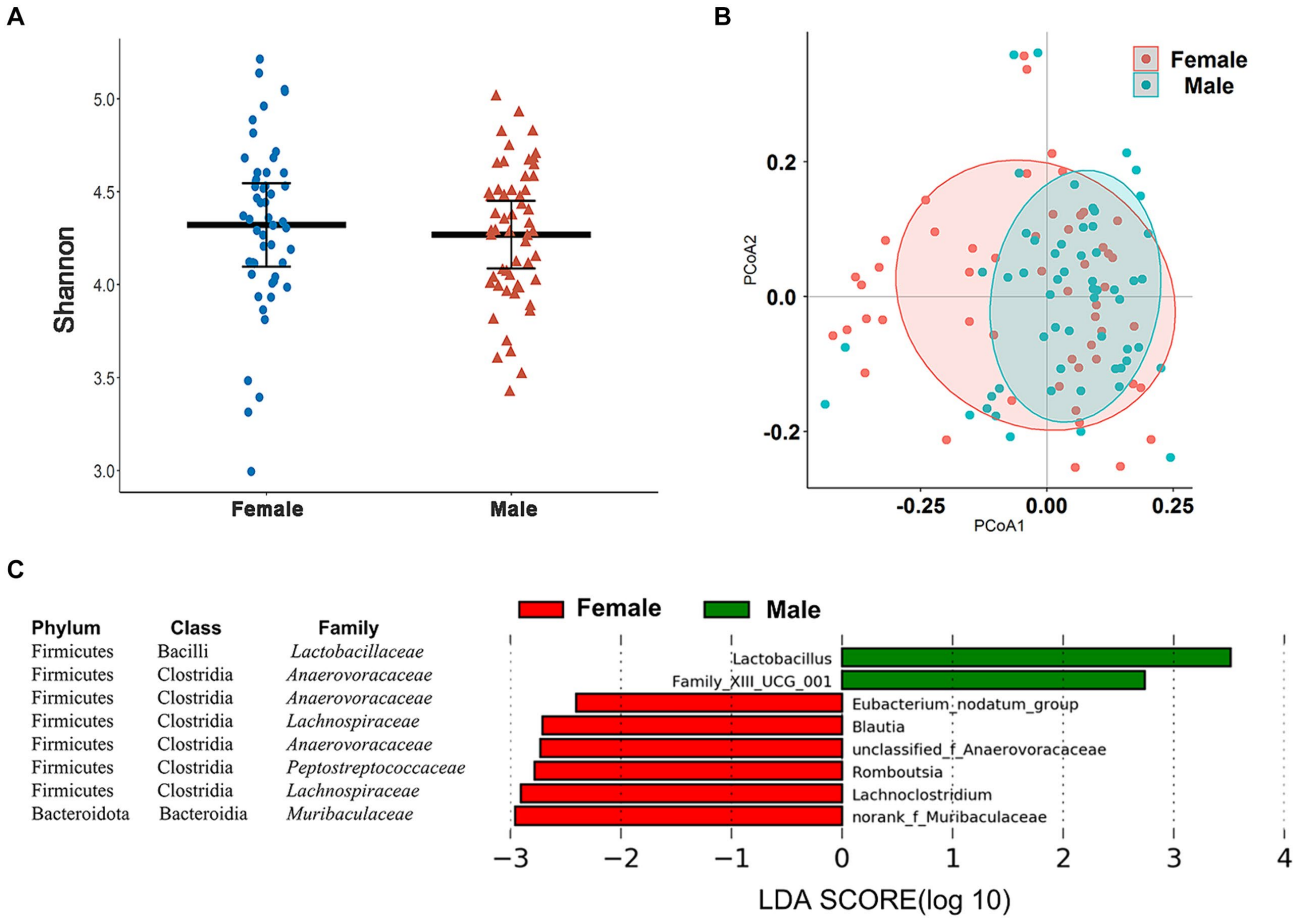
Sex-associated differences in cecal microbiome composition and diversity. **(A)** Comparison of the α-diversity (Shannon index) of cecal microbiota based on sex. **(B)** PCoA based on Bray–Curtis distances for the cecal microbiomes between male and female chickens. **(C)** Eight genera showing significantly different relative abundance levels between male and female chickens using LEfSe analysis.

In our taxonomic groupings, the Wilcoxon rank-sum tests and PCoA indicated the absence of gender differences for alpha and beta diversity of the cecal microbiome in these chickens (Figures 4A,B). We used LEfSe analysis at different taxonomic levels to determine whether we could identify specific taxa that displayed a gender bias. In the total cohort, no significant gender differences in bacterial phyla were detected (Supplementary Figure S2) although genera more abundant in male chickens included *Lactobacillus* and *Family_XIII_UCG_001* (LDA > 2.0, *p* < 0.05) while *Eubacterium_nodatum_ group*, *Blautia*, unclassified_Anaerovoraceae, *Romboutsia*, *Lachnoclostridium,* and norank_Muribaculaceae were more abundant in female chickens (Figure 4C).

### 3.4 Differences in serum metabolites between male and female chickens

**Figure 5:**
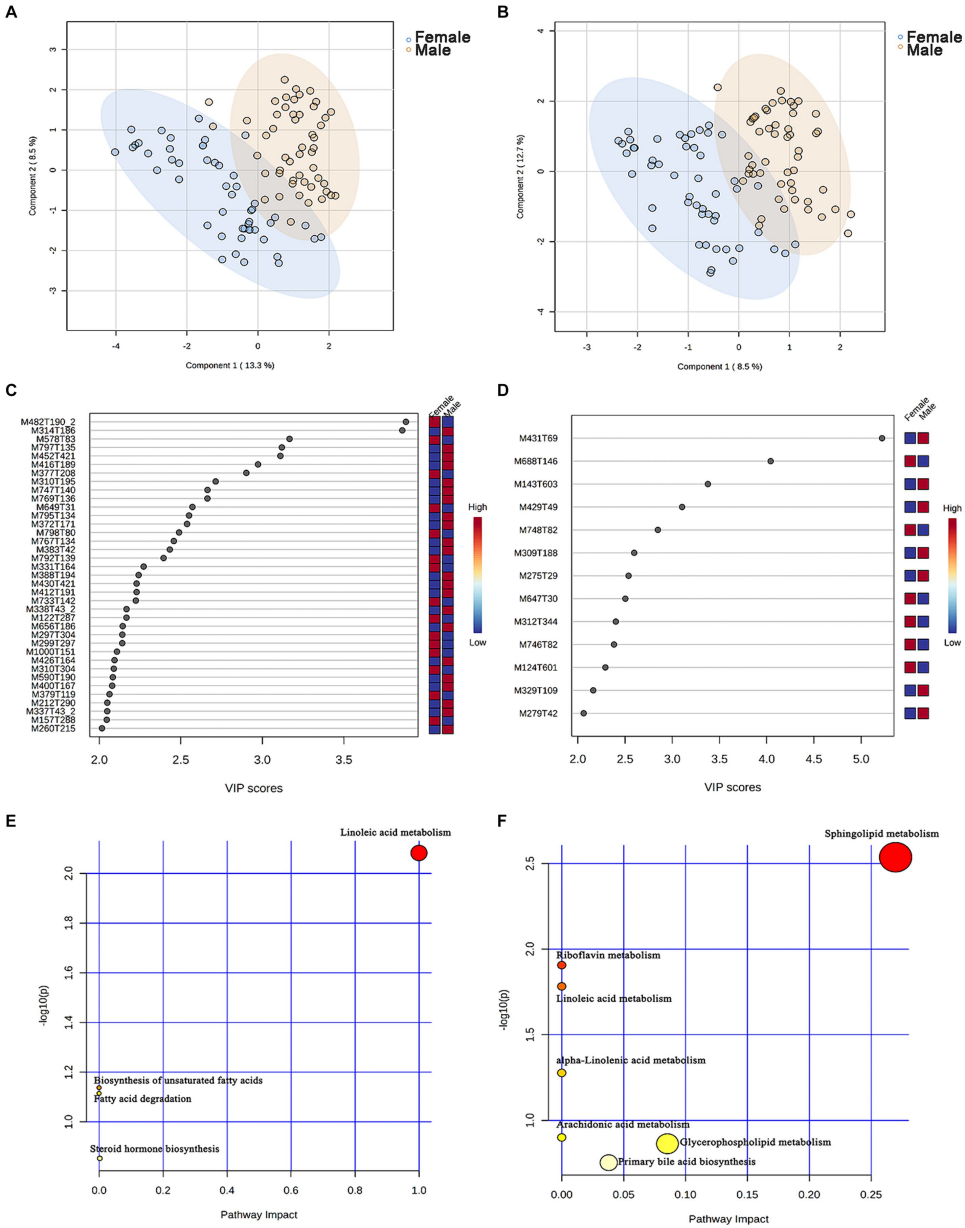
Identification of the metabolic signatures between male and female chickens. OPLS-DA of serum metabolomic data in **(A)** positive and **(B)** negative mode for male (*n* = 51, in blue) and female (*n* = 50, in brown) chickens. Variable importance in projection (VIP > 2) scores for the top serum metabolites in **(C)** positive and **(D)** negative mode contributing to variation in metabolic profiles of male and female chickens. The relative abundance of metabolites is indicated by a colored scale from blue to red representing the low and high, respectively. Pathway enrichment analysis based on metabolites associated with **(E)** male and **(F)** female chicken.

A non-targeted metabolomics analysis was performed on 101 chicken serum samples (male chickens *n* = 51, female chickens *n* = 50), 7,086 and 4,910 metabolite features were obtained in positive and negative modes, and 494 and 272 metabolites were annotated, respectively (Supplementary Tables S5, S6). An OPLS-DA model was constructed to identify differential serum metabolites between male and female chickens, and these plots displayed a clear separation within both positive and negative modes (Figures 5A,B).

**Figure 6:**
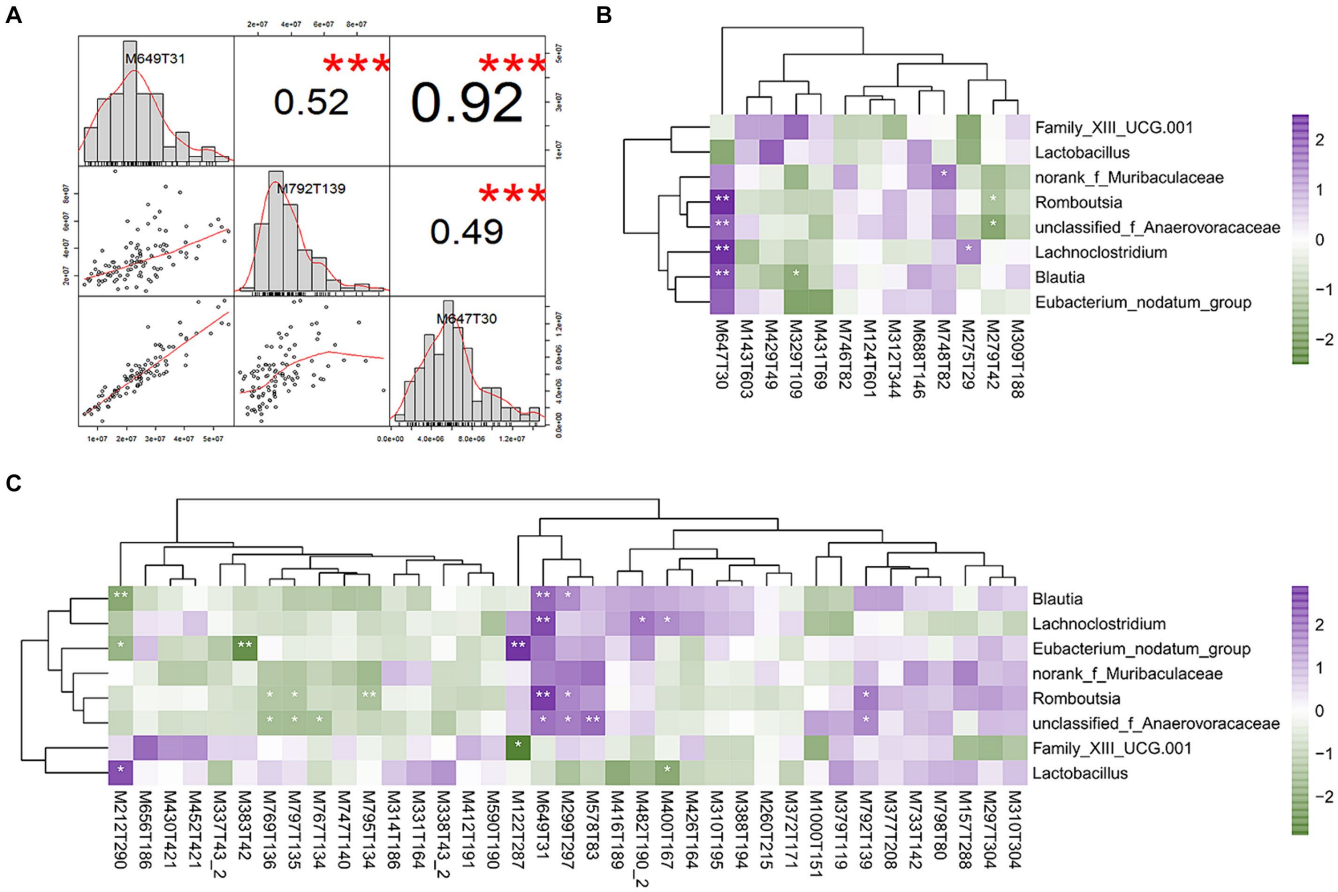
Correlations between differential serum metabolites and bacterial species. **(A)** Correlation of three sphingomyelin metabolites. **(B)** Heatmap depicting correlations between differential serum metabolites (negative mode) and differential bacterial species in difference sex chickens. **(C)** Heatmap depicting correlations between differential serum metabolites (positive mode) and differential bacterial species. *p < 0.05; **p < 0.01; and ***p < 0.001 were calculated using Spearman’s rank correlation test. Positive (in purple) and negative (in dark green) correlations are indicated.

To evaluate the differences in metabolic profiles between male and female chickens, we examined the patterns of specific metabolites. In the positive mode, 37 differential metabolites (VIP > 2, *p* < 0.05) were identified (Figure 5C; Table 2). In particular, 22 metabolites were enriched for male chickens and they included myristoyl-l-carnitine, prostaglandin F2α alcohol methyl ether, and Lys-Ile-Lys. Conversely, 15 metabolites were enriched in female chickens including riboflavin, taurochenodeoxycholic acid, and 3-hydroxy-3′,4′-dimethoxyflavone. In negative mode, a total of 13 metabolites showed significant differences between the male and female groups in which 7 metabolites were increased in the male chickens including 1β-hydroxytestosterone, prostaglandin F2β, and linoleic acid (Figure 5D; Table 3). Concentrations of 6 metabolites were significantly higher in female chickens and included phosphatidylethanolamine 32:1, phosphatidylglycerol 34:2, and N-(2-furoyl)glycine. It was worth noting that sphingomyelin metabolites contained N-tetracosenoyl-4-sphingenine (M649T31, positive mode), sphingomyelin (M792T139, positive mode), and N-[1,3-dihydroxyoctadec-4-en-2-yl]tetracos-15-enamide (M647T30, negative mode), were increased in female serum, and were significantly correlated with each other (Figure 6A). Among them, N-[1,3-dihydroxyoctadec-4-en-2-yl]tetracos-15-enamide and N-tetracosenoyl-4-sphingenine were most highly and significantly (*r* = 0.922, *P* < 0.001) correlated with female chickens. The correlation between sphingomyelin with N-tetracosenoyl-4-sphingenine and N-[1,3-dihydroxyoctadec-4-en-2-yl]tetracos-15-enamide was 0.515 and 0.491, respectively (*P* < 0.001). Three hormone-related metabolites (prostaglandin F2α alcohol methyl ether, prostaglandin F2β, and 11β-hydroxytestosterone) were enriched in male chicken serum. However, no hormone-related metabolites were identified in female chickens. We then performed a metabolic pathway analysis for the differential metabolites we identified above. The pathway enriched in male chickens was linoleic acid metabolism, while the sphingolipid metabolism pathway was enriched in female chickens (Figures 5E,F).

**Table 2:** Differences in serum metabolites between male and female chickens in positive ion mode.

ID	Enriched group	Annotated metabolites
M482T190_2	Female	1-hexadecyl-sn-glycero-3-phosphocholine
M578T83	Female	1-palmitoyl-2-oleoyl-sn-glycerol
M377T208	Female	(−)-riboflavin
M649T31	Female	N-tetracosenoyl-4-sphingenine
M798T80	Female	1,2-dioleoyl-sn-glycero-3-phospho-rac-1-glycerol
M792T139	Female	Sphingomyelin (d18:1/18:0)
M331T164	Female	Carnosol
M733T142	Female	1-oleoyl-2-myristoyl-sn-glycero-3-phosphocholine
M122T287	Female	Benzamide
M297T304	Female	Trigonelline
M299T297	Female	3-hydroxy-3′,4′-dimethoxyflavone
M1000T151	Female	Taurochenodeoxycholic acid
M310T304	Female	N-acetylneuraminic acid
M379T119	Female	Pyridate
M157T288	Female	N-.alpha.-acetyl-l-ornithine
M314T186	Male	1-methyl-2-undecylquinolin-4-one
M797T135	Male	Thioetheramide-PC
M452T421	Male	Doxazosin
M416T189	Male	Tomatidin
M310T195	Male	Metipranolol
M747T140	Male	1-hexadecyl-2-(9z-octadecenoyl)-sn-glycero-3-phosphocholine
M769T136	Male	1-hexadecyl-2-(5z,8z,11z,14z-eicosatetraenoyl)-sn-glycero-3-phosphocholine
M795T134	Male	1-(1z-octadecenyl)-2-(5z,8z,11z,14z-eicosatetraenoyl)-sn-glycero-3-phosphocholine
M372T171	Male	Myristoyl-l-carnitine
M767T134	Male	1-o-hexadecyl-2-o-(5z,8z,11z,14z,17z-eicosapentaenoyl)-sn-glyceryl-3-phosphorylcholine
M383T42	Male	Pinanethromboxane a2
M388T194	Male	Lys-Ile-Lys
M430T421	Male	N-(sec-butyl)-n-(4-(sec-butyl(trifluoroacetyl)amino)phenyl-2,2,2-trifluoroacetamide
M412T191	Male	Cyclopamine
M338T43_2	Male	N-cis-hexadec-9-enoyl-l-homoserine lactone
M656T186	Male	2-epahsa [dmed-fahfa]
M426T164	Male	Oleoyl-l-carnitine
M590T190	Male	3-alahpda [dmed-fahfa]
M400T167	Male	L-palmitoylcarnitine
M212T290	Male	Brimonidine
M337T43_2	Male	Prostaglandin f2.alpha. Alcohol methyl ether
M260T215	Male	Propranolol

**Table 3:** Differences in serum metabolites between male and female chickens in negative ion mode.

ID	Enriched group	Annotated metabolites
M688T146	Female	Pe 32:1
M748T82	Female	1-palmitoyl-2-oleoyl-phosphatidylglycerol
M647T30	Female	N-[1,3-dihydroxyoctadec-4-en-2-yl]tetracos-15-enamide
M312T344	Female	5-hydroxydiclofenac
M746T82	Female	Pg 34:2
M124T601	Female	N-(2-furoyl)glycine
M431T69	Male	Eplerenone hydroxy acid
M143T603	Male	Bisdemethoxycurcumin
M429T49	Male	(1-acetyloxy-3-hydroxy-6,8a-dimethyl-7-oxo-3-propan-2-yl-2,3a,4,8-tetrahydro-1 h-azulen-4-yl) 4-hydroxybenzoate
M309T188	Male	Prostaglandin f2.beta.
M275T29	Male	Methyl salicylate
M329T109	Male	11beta-Hydroxytestosterone
M279T42	Male	Linoleic acid

### 3.5 Correlation between sex-related metabolites and differential cecal microbes

We further established relationships between differential serum metabolites and differential cecal microbes based on gender using Spearman’s rank correlation analysis. The correlation coefficients were calculated for each pair of relative abundance of bacterial species and metabolites (Figures 6B,C; Supplementary Tables S7, S8). For sex hormone metabolites, 11β-hydroxytestosterone was enriched in male chickens and was significantly negatively correlated with *Blautia* (*r* = −0.217, *p* = 0.03). All three sphingomyelin metabolites were significantly positively correlated with a variety of cecal microbes that included *Blautia*, unclassified_Anaerovoraceae, *Romboutsia,* and *Lachnoclostridium*. Specifically, N-[1,3-dihydroxyoctadec-4-en-2-yl]tetracos-15-enamide was positively correlated with *Blautia* (*r* = 0.291, *p* = 0.003), unclassified_Anaerovoraceae (*r* = 0.267, *p =* 0.007), *Romboutsia* (*r* = 0.426, *p <* 0.001), and *Lachnoclostridium* (*r* = 0.347, *p <* 0.001). N-tetracosenoyl-4-sphingenine was positively correlated with *Blautia* (*r* = 0.266, *p* = 0.007), unclassified_Anaerovoraceae (*r* = 0.251, *p* = 0.011), *Romboutsia* (*r* = 0.360, *p* < 0.001), and *Lachnoclostridium* (*r* = 0.304, *p* = 0.002). Sphingomyelin was positively correlated with unclassified_Anaerovoraceae (*r* = 0.209, *p* = 0.036) and *Romboutsia* (*r* = 0.227, *p* < 0.022).

## 4 Discussion

### 4.1 Gender plays an important role in chicken production performance

Poultry production led by empirical evidence has traditionally set differing production systems based on gender. Sexual dimorphism was also associated with a variety of traits in chickens including growth rate, slaughter performance (increased muscle and decreased abdominal fat), feed conversion efficiency, and mineral utilization ability (Rose et al., 1996; Lumpkins et al., 2008; Cui et al., 2021; Tumova et al., 2021). Feed additives have demonstrated differential effects on animal health or production performance by gender (Han and Baker, 1993; Zhang D. et al., 2021). Therefore, gender, production performance, and nutrient utilization are linked and have important practical significance. However, the mechanisms that mediate these associations were poorly understood. A large body of data indicated clear contributions of gut microbiota or metabolites to these processes. We, therefore, conducted the current study to provide a research basis for sexual dimorphism in chicken production performance from the perspective of gut microbiota and metabolomics. We explored the differences in cecal microbiota and serum metabolome between male and female chickens and initially established the relationships between sex, the cecal microbiota, and serum metabolites.

### 4.2 Effect of gender on the diversity of cecal microbiota in chicken

Gender is an important factor affecting animal gut microbiota diversity (Zhang X. et al., 2021), and higher microbial diversity in female chickens has been demonstrated in studies of humans (Falony et al., 2016), pigs (Zhang D. et al., 2021), mice (Kozik et al., 2017), and chickens (Cui et al., 2021). In contrast, we found that cecal microbiota diversity did not differ significantly between male and female chickens. However, our network analysis indicated that sex could have a significant selection effect on cecal microbial interactions, and female chickens possessed a more complex and stable microbiome (Wang et al., 2021). Greater complexity and stability translate into greater resistance to the external environment (Coyte et al., 2015), and this implicates sexual traits with a significant selection effect on the microbiome. One possible reason for these effects is due to longer colon transit times in female chickens (Degen and Phillips, 1996; Graff et al., 2001), and longer colon transit times were associated with higher gut microbial complexity (Roager et al., 2016). Interestingly, previous studies in humans and other mammals have not consistently shown this (Haro et al., 2016; Gao et al., 2018; Takagi et al., 2019). However, reports of sex bias in the chicken gut microbiome have been inconsistent and may have been due to noise introduced by factors such as breed, diet, and feeding mode (Lee et al., 2017).

### 4.3 Enrichment of specific bacteria in the cecum of chickens of different genders

Prior chicken studies have reported the presence of characteristic microbiota for male and female chickens. Unlike these results of previous research results (Lee et al., 2017; Cui et al., 2021), we found that the abundance of *Lactobacillus* and *Family _XIII_UCG-001* was significantly higher in the cecal microbiota of male chickens, while *Eubacterium_nodatum_group*, *Blautia*, unclassified_Anaerovoraceae, *Romboutsia*, *Lachnoclostridium,* and norank_Muribaculaceae were more abundant in female chickens. Multiple previous studies have also confirmed that *Lactobacillus* is an important gender-differentiated microbe and its abundance in male chickens was significantly higher than for female chickens (Sha et al., 2013; Poutahidis et al., 2014; Sherman et al., 2018; He et al., 2019) and enrichment for *Lactobacillus salivarius*, *Lactobacillus crispatus*, and *Lactobacillus aviaries* was identified in male chickens (Torok et al., 2013). These studies indicated that *Lactobacillus* may be an important gut microbe relevant to male chickens. The presence of *Lactobacillus* in human or animal males has also been linked to inhibition of inflammation and increased testosterone levels (Poutahidis et al., 2014). Dietary supplementation with probiotic *Lactobacillus reuteri* could prevent age-related testicular atrophy in mice and male hypogonadism in humans (Cross et al., 2018). Conversely, studies in mice have reported a different pattern where females exhibited a more abundant presence of *Lactobacillus* in their bacterial community, which was linked to their stronger immunological response to pathogenic bacteria infection than males (Ge et al., 2006). We found that *Blautia* was enriched in the ceca of female chickens and was consistent with human studies that linked *Blautia* with adult women (Markle et al., 2013). *Eubacterium* in pigs has also been reported to be sex-differentiated (He et al., 2019). However, unclassified_Anaerovoraceae, *Romboutsia*, *Lachnoclostridium*, and norank_Muribaculaceae (this study) have not been previously reported.

### 4.4 Differences in serum metabolome of chickens from different genders

Metabolome analysis was carried out in the current study, revealing clear differences in serum metabolites between male and female chickens. Three sex hormones were enriched in male chicken serum (prostaglandin F2α and its alcohol methyl ether isomer and 11β-hydroxytestosterone), while no characteristic steroid hormone metabolites were identified in female chickens. Male chickens in this study were 18 weeks old and had reached sexual maturity, and this is most likely the reason for these results. In addition, we identified other serum metabolites showing significant differences by gender and included three sphingomyelin metabolites (N-tetracosenoyl-4-sphingenine, sphingomyelin, and N-[1, 3-dihydroxyoctadec-4-en-2-yl] tetracos-15-enamide) that were enriched in female chickens. Metabolite pathway enrichment analysis also verified that the sphingolipid metabolism pathway was the primary metabolic pathway enriched in female chicken, sera and sphingomyelin is essential for egg yolk formation (Yang et al., 2018). Sex hormones are the primary causes of sexual dimorphism in animals (Degen and Phillips, 1996; Org et al., 2016; Lee et al., 2017). Chickens in our study were already mature sexually, and some began to lay eggs. Female chickens began to shift from growth and development to reproduction and gradually prepared for the formation of follicles (Kim et al., 2020), and this might be the reason for the elevated levels of sphingomyelin metabolites we found. In addition, linoleic acid was enriched in male sera, and this result was also verified in metabolic pathway analysis. Linoleic acid could change the structure of cumulus granulosa cell membranes and interfere with gonadotropins thereby inhibiting oocyte maturation (Prades et al., 2003). Our female chickens were physiologically prepared for laying eggs with associated elevated estrogen levels, and this could exert a negative (inhibitory) effect on linoleic acid production.

### 4.5 Sex-specific cecal bacteria participate in the metabolic process of serum metabolites

Since the gut microbiota was involved in the excretion and circulation of sex hormones, researchers have proposed the concept of a microgenderome to characterize the microbiome associated with sex hormone metabolism (Yoon and Kim, 2021). In our study, we identified numerous significant pairs and intensity of correlation, and some serum metabolites displayed strong correlations with the cecal microbiomes. A correlation analysis was conducted between gender-differential sex hormone metabolites in serum and gender-differential microbiota, and *Blautia* and 11β-hydroxytestosterone displayed a significant negative correlation. *Blautia* has been reported as an important characteristic genus in women (Markle et al., 2013). Notably, we also found that three sphingomyelin metabolites were positively correlated with *Blautia*, unclassified_Anaerovoraceae, *Romboutsia,* and *Lachnoclostridium.* These associations suggested that the cecal microbiota may be involved in the synthesis or metabolism of these sphingolipid metabolites or that these metabolites had a role in regulating the composition of the chicken cecal microbiome. Humans possess numerous sphingolipid-producing bacteria including *Bacteroides* and *Parabacteroides* (Gupta and Lorenzini, 2007; Heaver et al., 2018). Sphingomyelin was also linked to the regulation of the abundance of specific microbes in the intestine. For example, 1% purified glycosylceramide (N-[1,3-dihydroxyoctadec-4-en-2-yl]tetracos-15-enamide, this study) fed continuously to mice for 1 week increased the abundance of *Blautia coccoides*, and this species could degrade glycosylceramide into ceramide that female chickens can metabolize into fatty acids and sphingosine that could be absorbed by the intestine and exert beneficial effects on the host (Hamajima et al., 2016). In addition, unclassified_Anaerovoraceae, *Romboutsia*, and *Lachnoclostridium* have not been previously reported to be linked with host serum sphingomyelin metabolism (Hannun and Obeid, 2018; Heaver et al., 2018). Together with our study, these reports point to an important causal relationship between cecal microbes and sphingomyelin metabolites, and this extends to the female chicken microbiome.

## 5 Conclusion

Our results support the role of sex differences in shaping cecal microbial communities in chickens, and we identified *Lactobacillus*, *Family_XIII_UCG-001*, *Eubacterium_nodatum_group*, *Blautia*, unclassified_Anaerovoraceae, *Romboutsia*, *Lachnoclostridium*, and norank_Muribaculaceae as important gender-related microbes. Androgen and sphingomyelin metabolites appear to be responsible in part for these sex differences, and specific cecal microbes were closely related to the levels of some of these types of serum metabolites. Revealing these interactions between the cecal microbiome and the serum metabolome by gender could ultimately lead to the identification of novel factors that influence production performance and improve diagnostic and precision feed design.

However, although this study initially established the relationship between gender, the microbiome, and the metabolome, there were also some limitations that should be addressed or avoided in subsequent studies. First, our study only compared the differences between cecal microbiota and serum metabolome but not in conjunction with economic traits. Second, this study focused more on establishing the association among sexual dimorphism, cecal microbiota, and metabolites. The causal relationship among them needs further experimental confirmation. Therefore, future research should combine specific economic traits and explore the impact of chicken gender differences on the trait formation mechanism by experimental verification.

## Data availability statement

The datasets presented in this study can be found in online repositories. The names of the repository/repositories and accession number(s) can be found in the article/Supplementary material.

## Ethics statement

All animal works were conducted according to the guidelines for the care and use of experimental animals established by the Ministry of Agriculture and Rural Affairs of China. Animal Care and Use Committee in Guizhou University specially approved this project (No.EAE-GZU-2022-T050). The study was conducted in accordance with the local legislation and institutional requirements.

## Author contributions

YY: Investigation, Methodology, Visualization, Writing – original draft. FZ: Data curation, Investigation, Resources, Software, Writing – original draft. XY: Investigation, Methodology, Writing – original draft. LW: Data curation, Validation, Writing – original draft. ZW: Conceptualization, Funding acquisition, Project administration, Supervision, Writing – review & editing.

## Footnotes

^1^Release 132, http://www.arb-silva.de.

^2^ https://qiime2.org/

^3^ http://huttenhower.sph.harvard.edu/galaxy

^4^ https://www.metaboanalyst.ca

## Supplementary material

SUPPLEMENTARY FIGURE S1 Chicken sex is significantly associated with chicken cecal microbiome composition.

SUPPLEMENTARY FIGURE S2 Relative abundance of bacterial phyla associated with sex.
