# Supplementary figures and images for: Integrating microbial 16S rRNA sequencing and non-targeted metabolomics to reveal sexual dimorphism of the chicken cecal microbiome and serum metabolome
Source: Front Microbiol. 2024 Jul 19;15:1403166. doi: 10.3389/fmicb.2024.1403166 (PMC11294938; doi:10.3389/fmicb.2024.1403166)

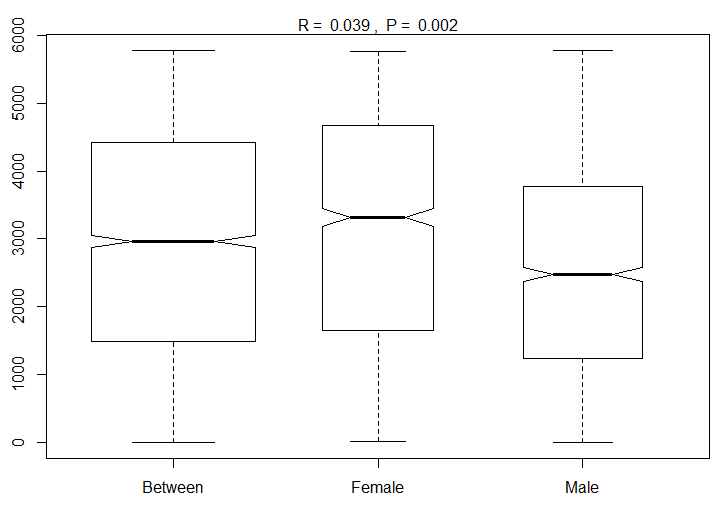

Supplement: SUPPLEMENTARY FIGURE S1 — Chicken sex is significantly associated with chicken cecal microbiome composition. [file Image_1.TIFF]

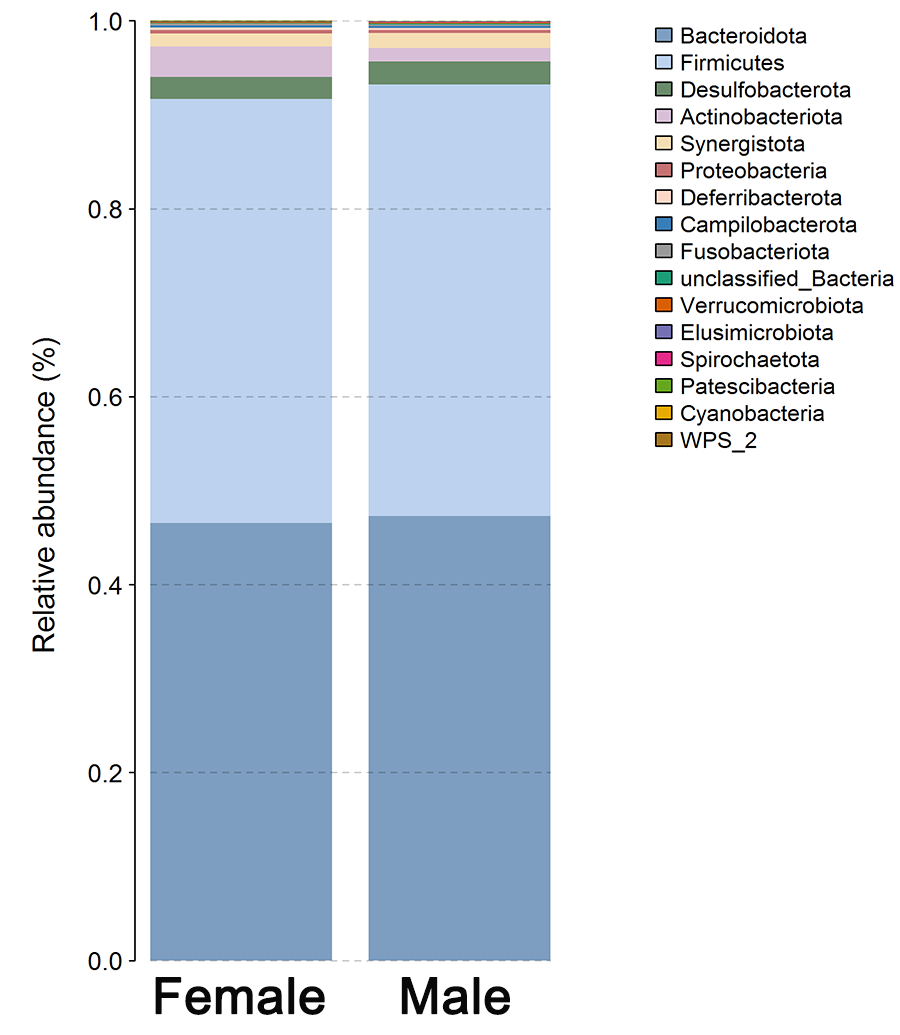

Supplement: SUPPLEMENTARY FIGURE S2 — Relative abundance of bacterial phyla associated with sex. [file Image_2.tif]

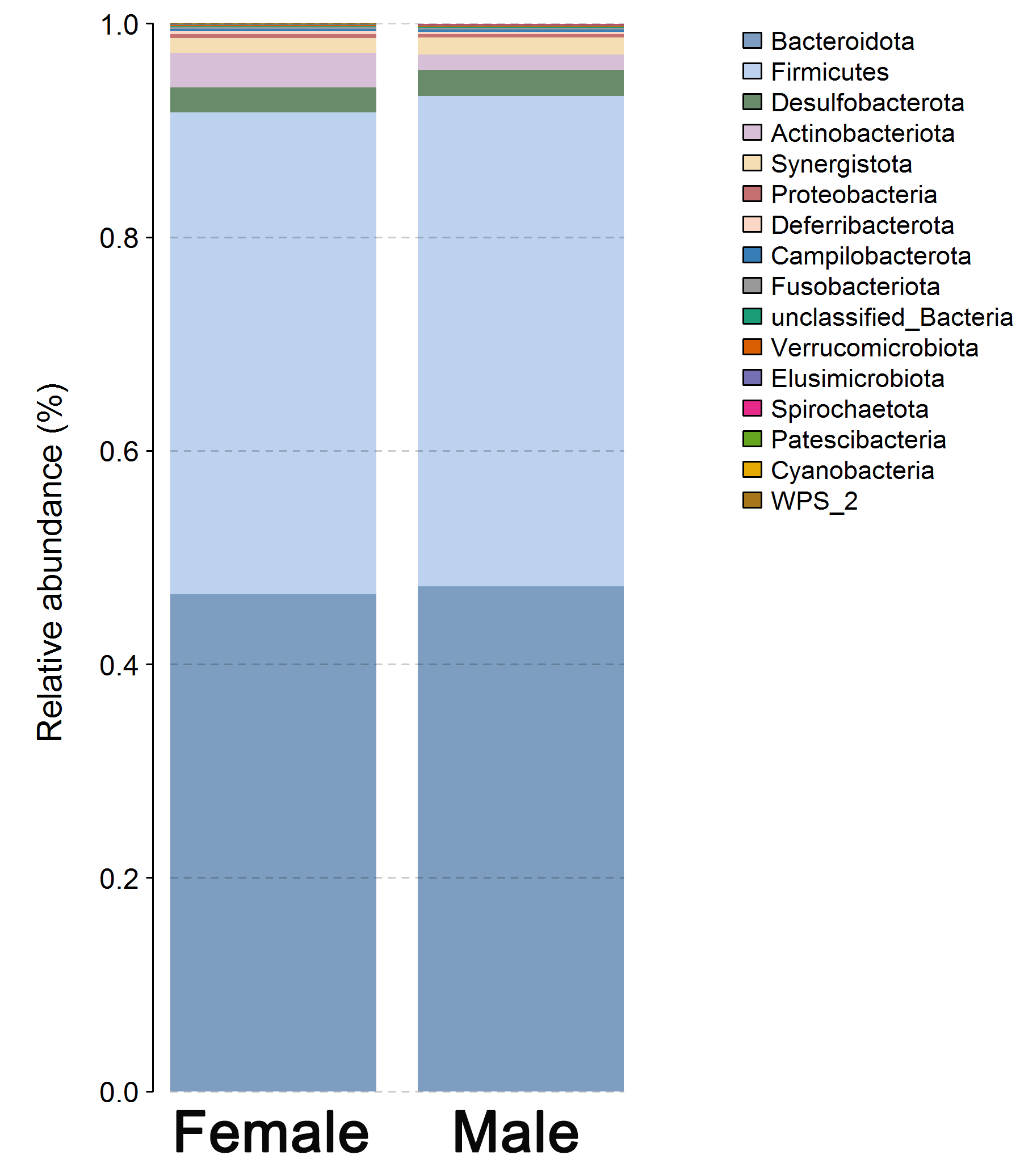

Supplement: Supplementary file 3 [file Image_3.TIF]
