# Supplementary material for: Integrating microbial 16S rRNA sequencing and non-targeted metabolomics to reveal sexual dimorphism of the chicken cecal microbiome and serum metabolome
Source: Front Microbiol. 2024 Jul 19;15:1403166. doi: 10.3389/fmicb.2024.1403166 (PMC11294938; doi:10.3389/fmicb.2024.1403166)
Supplement: Supplementary file 5 [file Table_2.docx]

**Supplementary Table S2** Interaction effects between breed and sex analyzed by two-way ANOVA

| **Source** | **Type III Sum of Squares** | **df** | **Mean Square** | **F** | **p** |
| --- | --- | --- | --- | --- | --- |
| Intercept | 3570.793 | 1 | 3570.79 | 8291.688 | 0.000 |
| Breed | 29.000 | 1 | 29.000 | 67.343 | 0.000 |
| Sex | 46.770 | 1 | 46.770 | 108.605 | 0.000 |
| Breed * Sex | 0.073 | 1 | 0.073 | 0.170 | 0.681 |
| Corrected Total | 117.295 | 105 | 0.430 |  |  |

R Squared=0.626(Adjusted R Squared=0.614)
